# Supplementary material for: Nonlinear control of transcription through enhancer–promoter interactions
Source: Nature. 2022 Apr 13;604(7906):571–7. doi: 10.1038/s41586-022-04570-y (PMC9021019; doi:10.1038/s41586-022-04570-y)
Supplement: Supplementary file 1 — Model description and gating strategy. [file 41586_2022_4570_MOESM1_ESM.pdf]

nature portfolio

https://doi.org/10.1038/s41586-022-04570-y

Supplementary information

Nonlinear control of transcription through enhancer–promoter interactions

In the format provided by the authors and unedited

Supplementary Information: Nonlinear control of transcription through enhancer-promoter interactions

Jessica Zuin, Gregory Roth,..., Luca Giorgetti

Supplementary Information Guide

Supplementary Model Description, p.1 to 15

Gating strategy, p. 16 to 21

Supplementary Model Description for Nonlinear control of transcription through enhancer-promoter interactions

Jessica Zuin, Gregory Roth,..., Luca Giorgetti

Contents

1 Two-state model 1

2 Variable two-state models 2
2.1 Model selection 2

3 Variable k_{on} two-state model fitting 3
3.1 RNA FISH data 3
3.2 Mean eGFP levels 4
3.3 Model fitting for the full-length SCR data set 4
3.4 Profile likelihood analysis and confidence interval 4

4 Mechanistic model of enhancer-promoter communication 5
4.1 Model description 5
4.2 Mean and variance of the number of RNA 7
4.3 Mean and variance of the number of RNAs as a function of the contact probability 7

5 Qualitative study of the transcriptional response to changes in contact probabilities 8
5.1 Reduction of the enhancer-promoter model to an apparent two-state model . . . 8
5.2 Sigmoidallity of the apparent *on* rate 8

6 Enhancer-promoter model fitting 10
6.1 Full SCR data set 11
6.2 Truncated SCR data set 11

In this Supplementary Information we describe the different mathematical models used in the study, their analysis and the fitting procedure.

1 Two-state model

The two-state model of gene expression (first introduced by Peccoud and Ycart [1995]) describes the promoter as stochastically switching between an *off* state (inactive) and an *on* state (active) where transcription can occur. The promoter transitions between those states with rate

k_{on} and k_{off}. Synthesis and degradation of RNAs is regarded as a Poisson process (i.e. we describe RNA initiation, elongation, nuclear export of RNA as a single kinetic step) and occur at rates μ and δ, respectively. In this model, the mean and variance of the number of mRNAs are given by the following formulas

$$\langle \text{RNA} \rangle = \frac{k_{on}}{k_{on} + k_{off}} \frac{\mu}{\delta} \tag{1}$$

$$\text{Var}(\text{RNA}) = \langle \text{RNA} \rangle + \frac{k_{on} k_{off}}{(k_{on} + k_{off})^2} \frac{\mu^2}{\delta(k_{on} + k_{off} + \delta)} \tag{2}$$

(Peccoud and Ycart [1995]). Although there exists an analytical expression of the steady-state distribution of the number of mRNAs, its computation is difficult because it requires the calculation of the confluent hypergeometric function for which there is no general fast numerical method for its computation. Here, we calculate the steady-state probability distribution of the two-state model by using the finite state projection algorithm for the stationary solution of the chemical master equation(Gupta et al. [2017]). This method consists of truncating the infinite state space of the system into a finite subset of states in order to reduce the infinite-dimensional system of ODEs into a finite system. For the two-state model, this truncation consists in fixing a maximal number of RNAs per cell which we set to 120% of the maximal number observed in the FISH experiment. The code for these calculations was written in Matlab (version 2019b) and are available on Github (`https://github.com/gregroth/Zuin_Roth_2021`).

2 Variable two-state models

Based on the observation that the mean number of mRNAs per cell increases nonlinearly with the contact probability between the promoter and its enhancer (Fig. 2B in the main text), we asked if this nonlinearity could be reproduced by a *variable two-state model* in which one of its rate depends nonlinearly on the contact probability. We define 3 variable two-state models: the variable k_{on} two-state model for which the *on* rate depends on contact probability, the variable k_{off} two-state model for which the *off* rate depends on contact probability, and the variable μ two-state model for which the initiation rate depends on contact probability. For each of these models, the dependency of the variable parameter on contact probability is described by a Hill function. For example, in the variable k_{on} two-state model, we model the *on* rate as

$$k_{on}(p_c) = k_{on}^0 + \frac{p_c^h}{c + p_c^h} \left(k_{on}^0 - k_{on}^1 \right). \tag{3}$$

where k_{on}^0 and k_{on}^1 correspond to the lowest and the highest values, respectively, of the *on* rate, and the Hill exponent h and the parameter c control the "type" of nonlinear dependency. When h is smaller than 1, the variable parameter is a sublinear function of contact probability. When h is larger than 1 and c is smaller than 1, the variable parameter is a sigmoidal-like (i.e. it has an inflexion point at an intermediate value of contact probability).

2.1 Model selection

Although the 3 variable two-state models are able to reproduce the nonlinear transcriptional response observed in Fig. 2B, only the variable k_{on} two-state model can reproduce the cell-to-cell variability measured in the 6 smRNA FISH experiments. For each smRNA FISH distribution

we calculated, Δ, the difference between the squared coefficient of variation and the inverse of the mean which provides a measure of how the smRNA FISH distribution deviates from a Poisson distribution ($\Delta = 0$ for a Poisson distribution). We observed that Δ steeply decreases when the contact probability increases (see Figure 1 in this document). We now show that the μ two-state model and the k_{off} two-state model can not reproduce this observed steep decrease in Δ. From equations (1) and (2), we deduce the deviation Δ in a two-state model,

$$\Delta = \frac{k_{off}}{k_{on}} \frac{\delta}{(k_{on} + k_{off} + \delta)}. \tag{4}$$

First, it is clear that the variable μ two-state model can not account for the smRNA FISH data because Δ does not depends on μ. Second, we note from equation (4) that $\Delta < \frac{1}{k_{on}}$ and we also note that the Δ calculated for the clone corresponding to contact probability 0 is equal to 9 (Figure 1 in this document). Taken together this means that the rate k_{on} in a variable k_{off} two-state model should be smaller than 1 which contradicts the fact that we observe unimodal distributions for clone associated with high contact probabilities (Fig. 2D). Indeed, it has been shown that a two-state model with an *on* rate smaller than the degradation rate shows either bimodal distribution or long distribution tails Munsky et al. [2012].

3 Variable k_{on} two-state model fitting

The variable k_{on} two-state model described above was fitted simultaneously to the mean eGFP levels measured in individual cell lines and to the distributions of RNA numbers measured by smRNA FISH in 6 cell lines where the full-length Sox2 control region (SCR) was located at different distances from the promoter. For each cell line C^k (i.e.$k = 1, \dots, 6$), we note p_{c_k} the measured contact probability between the enhancer and the promoter. This section describes in detail how this was done.

In the sequel, all the rates are expressed in unit of 1 over the mean life time of a mRNA molecule (i.e. we set $\delta = 1$).

3.1 RNA FISH data

For each cell line C^k (i.e.$k = 1, \dots, 6$), we calculated the histogram, $\mathbf{h}^k$ of the RNA molecule counts obtained from the smRNA FISH experiment. The bin size b_k and the number of bins n_b^k were chosen using the function *histogram* in Matlab.

For each set of parameters $\boldsymbol{\theta} = (k_{on}^0, k_{on}^1, k_{off}, \mu, c, h)$ we calculated the steady-state probability distributions of the two-state model with parameters $(k_{on}(p_{c_k}), k_{off}, \mu)$, where $k_{on}(p_{c_k})$ is given by equation (3). Next, we discretised the steady-state distributions in a histogram $\boldsymbol{\eta}^k(n, \boldsymbol{\theta})$ which is comparable with the histogram $\mathbf{h}^k$ obtained from the FISH data. We assume that the count in bin i for cell line k follows a binomial distribution of mean $\eta_i^k N_k$ and variance $\eta_i^k(1 - \eta_i^k)N_k$, where N_k is the total number of counts. We approximate the binomial distribution by a normal distribution. Hence, the likelihood of observing the histogram $\mathbf{h}^k$ given the parameters $(n, \boldsymbol{\theta})$ is

$$L_k(\boldsymbol{\theta}) = \prod_{i=1}^{n_b^k} \frac{1}{\sqrt{2\pi N_k \eta_i^k (1 - \eta_i^k)}} e^{-\frac{(h_i^k - \eta_i^k(\boldsymbol{\theta}))^2}{2\eta_i^k (1-\eta_i^k) N_k}} \tag{5}$$

and the log likelihood is

$$LL_k(\boldsymbol{\theta}) = \sum_{i=1}^{n_b^k} \left[-\frac{(h_i^k - \eta_i^k(\boldsymbol{\theta}))^2}{2\eta_i^k(1-\eta_i^k)N_k} + \frac{1}{2}\log(2\pi N_k \eta_i^k(1-\eta_i^k)) \right] \quad (6)$$

3.2 Mean eGFP levels

The data consist of the inferred mean number of RNA molecule per cell in each of the N individual eGFP+ cell lines (obtained via the calibration with sRNA FISH (Suppl. Fig. 1H)) and the associated genomic distance from the promoter to the SCR. The data were then averaged in bins of length 20 kb, yielding a vector $\mathbf{g}$ whose elements are the binned mean number of RNA per cell and a vector $\boldsymbol{d}$ of genomic distances. The genomic distances were transformed in contact probabilities using the Capture Hi-C data (6.4-kb resolution; see Figure 2A), yielding a vector $\boldsymbol{\pi}$ of contact probabilities associated to the vector $\boldsymbol{g}$.

For each set of parameters $\boldsymbol{\theta} = (k_{on}^0, k_{on}^1, k_{off}, \mu, c, h)$, and each cell line i, we calculate the mean number of RNA per cell, $\gamma_i(n, \boldsymbol{\theta})$, predicted by the two-state model with parameter $(k_{on}(p_{c_k}), k_{off}, \mu)$, where $k_{on}(p_{c_i})$ is given by equation (3). This mean was calculated using equation (1). Assuming that the deviations from the model are normally distributed with mean 0 and variance σ^2, the log likelihood function is given by

$$LL_{mean}(\boldsymbol{\theta}) = -\sum_{i=1}^{N} \frac{(g_i - \gamma_i(\boldsymbol{\theta}))^2}{2\sigma^2} + N\frac{1}{2}\log(2\pi\sigma) \quad (7)$$

where N is the number of cell lines.

3.3 Model fitting for the full-length SCR data set

We fit the variable k_{on} two-state model simultaneously to both the binned mean eGFP levels measured in individual cell lines and the RNA FISH distributions. The best fit parameter maximises the total log likelihood function

$$LL_{tot} := LL_{mean}(\boldsymbol{\theta}) + \sum_{k=1}^{6} LL_k(\boldsymbol{\theta}). \quad (8)$$

We set a lower bound of 0 for all the parameters and an upper bound of 1000 for the parameters $k_{on}^0, k_{on}^1, k_{off}, \mu$, an upper bound of 1 for the parameter c and an upper bound of 10 for the parameter h. We ensured that the best fit parameters found were not at the boundaries. For all the maximisations we use a global search approach. Specifically, we use the Matlab function *MultiStart* in the *Global Optimization* toolbox. All codes were written in Matlab (version 2019b) and are available at `https://github.com/gregroth/Zuin_Roth_2021`.

3.4 Profile likelihood analysis and confidence interval

We calculated the profile likelihood of all the parameters and derived their confidence intervals (see e.g. Pawitan [2001]). Let us denote $\boldsymbol{\theta} = (k_{on}^0, k_{on}^1, k_{off}, \mu, c, h)$ such that θ_j corresponds to the jth parameter (e.g. θ_3 corresponds to k_{off}). The profile likelihood function of parameter j is

$$PL_j(x) = \max_{\boldsymbol{\theta}|\theta_j = x} LL_{tot}(\boldsymbol{\theta}), \quad (9)$$

i.e. for each value x of parameter j the log-likelihood is maximised over the other parameters. The 95% confidence interval of parameter j is

$$CI_j = \{x | LL_{tot}(\boldsymbol{\theta}^*) - PL_j(x) \leq 3.8415\} \tag{10}$$

where $\boldsymbol{\theta}^* = \arg\max_{\boldsymbol{\theta}} LL_{tot}(\boldsymbol{\theta})$ and 3.8415 is the .95-quantile of the chi squared distribution with one degree of freedom. The profile likelihood functions were estimated using the Matlab function *MultiStart* in the *Global Optimization* toolbox. The plots of the profile likelihood functions are shown in Fig. S3 B.

4 Mechanistic model of enhancer-promoter communication

4.1 Model description

The mechanistic model of enhancer-promoter communication described in the main text is fully stochastic and describes the time evolution of four variables: the enhancer state, the promoter state, the promoter regime state, and the number of RNA molecules per cell. The enhancer states represent the relative position of the enhancer and the promoter (e_1 := close: the enhancer is in physical proximity of the promoter, i.e. their distance is smaller than an arbitrary threshold; e_2 := far: the enhancer is not in physical proximity of the promoter). The promoter states describe the transcriptional activity of the promoter (s_1 = off: the promoter cannot initiate transcription; s_2 := on: the promoter is prone to initiate transcription). In addition there are $n+1$ "promoter regime" states, which we divide in two sets: $\{r_1, \ldots, r_n\}$ describe the *basal* two-state promoter regime and the state r_{n+1} describes the *enhanced* two-state promoter regime. Transitions through the "promoter regime" states represent the regulatory processes that transmit regulatory information from the enhancer to the promoter. The promoter remains in the basal regime until all the n regulatory processes have been completed, and only at that point it can transition into the enhanced regime (see Figure 2 in this document). The basal and enhanced regimes differ only in their *on* rate. Finally, the number of RNA molecules per cell can be any integer $m \geq 0$.

We assume that the enhancer switches between its close and far states independently of the promoter and the promoter regime state. Transitions among the promoter regime states are reversible, however a forward transition is only possible when the enhancer is in the close state. Transition between the *on* and *off* state of the promoter are reversible. The *on* rate depends on the promoter regime while the *off* rate is the same for both regimes. Transcription can only be initiated from the promoter state s_2 (i.e *on* state, either in the basal or enhanced regime). Synthesis and degradation of RNAs is regarded as a Poisson process (i.e. we describe RNA initiation, elongation, nuclear export of RNA as a single kinetic step) and are both independent on the promoter regime. Note that "$n+1$ promoter regime states" actually means that there are n intermediate regulatory steps required to enter the enhanced regime. The kinetic reactions

are as follows.

$$
\begin{array}{rcl}
e_1 & \underset{k_{close}}{\overset{k_{far}}{\rightleftharpoons}} & e_2 \\
e_1 + r_k & \underset{k_{back}}{\overset{k_{forward}}{\rightleftharpoons}} & e_1 + r_{k+1}, \text{ for } \ k = 1, \dots, n \\
e_2 + r_k & \underset{k_{back}}{\leftarrow} & e_2 + r_{k+1}, \text{ for } \ k = 1, \dots, n \\
s_1 + r_k & \underset{k_{off}}{\overset{k_{on}^{basal}}{\rightleftharpoons}} & s_2 + r_k, \text{ for } \ k = 1, \dots, n \\
s_1 + r_{n+1} & \underset{k_{off}}{\overset{k_{on}^{enh}}{\rightleftharpoons}} & s_2 + r_{n+1} \\
m + s_2 + r_k & \underset{\mu}{\rightarrow} & m + 1 + s_2 + r_k, \text{ for } \ k = 1, \dots, n+1 \\
m & \underset{\delta}{\rightarrow} & m - 1
\end{array}
$$

The chemical master equation is given by

$$
\begin{aligned}
\frac{dp}{dt}(e_i, s_j, r_k, m) &= (m+1)\delta p(e_i, s_j, r_k, m+1) + \mu_{(i,j,k)} p(e_i, s_j, r_k, m-1) \qquad (11) \\
&\quad + \sum_{(\bar{i},\bar{j},\bar{k})} k_{(\bar{i},\bar{j},\bar{k};i,j,k)} p(e_i, s_j, r_k, m) \qquad (12) \\
&\quad - (m\delta + \mu_{(i,j,k)} + \sum_{(\bar{i},\bar{j},\bar{k})} k_{(i,j,k;\bar{i},\bar{j},\bar{k})}) p(e_i, s_j, r_k, m) \qquad (13)
\end{aligned}
$$

where $\mu_{(i,j,k)}$ is the transcription rate given the enhancer state, promoter state and the promoter regime state, and $k_{(i,j,k;\bar{i},\bar{j},\bar{)}}$ is the transition rate for the enhancer and promoter to go from the states e_i, s_j, r_k to the states $e_{\bar{i}}, s_{\bar{j}}, r_{\bar{k}}$. All rates are expressed in terms of the model parameters and are defined in Table 1.

This model can be rephrased in the general framework of multi-state promoter models (Sánchez and Kondev [2008]) if we interpret the triplet (e, s, r) as a "hyper" promoter state. There are $4(n + 1)$ hyper promoter states which can be described either by a triplet (i, j, k) where e_i defines the enhancer state, s_j defines the promoter state and r_k defines the promoter regime state, or it can be described by an integer $\varphi \in \{1, 2, \dots, 4(n+1)\}$. The two descriptors are connected by the so-called linear indexing bijection

$$
(i, j, k) \rightarrow \varphi = f(i, j, k) := (i - 1)2(n + 1) + (j - 1)(n + 1) + k. \qquad (14)
$$

Using this new notation, the chemical master equation can be rewritten as

$$
\begin{aligned}
\frac{dp}{dt}(\varphi, m) &= (m+1)\delta p(\varphi, m+1) + \mu_\varphi p(\varphi, m-1) \\
&\quad + \sum_{\vartheta} k_{(\vartheta;\varphi)} p(\varphi, m) \\
&\quad - (m\delta + \mu_\varphi + \sum_{\vartheta} k_{(\varphi;\vartheta)}) p(\varphi, m)
\end{aligned}
$$

Following Sánchez and Kondev [2008], we define the probability vector

$$
\mathbf{p}(m) = [p(1, m), \dots, p(4(n + 1), m)]
$$

and rewrite the chemical master equation in matrix form

$$\frac{d\mathbf{p}}{dt}(m) = (\mathbf{K} - \mathbf{T} - m\mathbf{\Delta})\mathbf{p}(m) + (m+1)\mathbf{\Delta}\mathbf{p}(m+1) + \mathbf{T}\mathbf{p}(m-1) \tag{15}$$

where the matrix $\mathbf{K}$ has elements $K_{\varphi\vartheta} = k_{(f^{-1}(\vartheta);f^{-1}(\varphi))}$ if $\varphi \neq \vartheta$ and $K_{\varphi\varphi} = -\sum_{\vartheta\neq\varphi} K_{\varphi\vartheta}$; the matrix $\mathbf{T}$ is diagonal with diagonal elements $T_{\varphi\varphi} = \mu_{f^{-1}(\varphi)}$; and the matrix $\mathbf{\Delta}$ is also diagonal with diagonal elements $\mathbf{\Delta}_{\varphi\varphi} = \delta$. The matrix $\boldsymbol{K}$ is the transition rate matrix of the Markov chain describing the stochastic dynamics of the enhancer-promoter interaction, the intermediate regulatory steps, and the promoter *on/off* switch. The steady-state probability distribution of the Markov chain $\boldsymbol{K}$ is the solution (subject to normalisation) of the equation

$$\boldsymbol{K}\boldsymbol{v} = 0. \tag{16}$$

4.2 Mean and variance of the number of RNA

The mean and the variance of the number of RNAs per cell is given by the following formulas (Sánchez and Kondev [2008])

$$\langle \text{RNA} \rangle = \frac{\boldsymbol{\mu}\boldsymbol{m}^{(0)}}{\delta} \tag{17}$$

$$\text{Var}(\text{RNA}) = \frac{\boldsymbol{\mu}\boldsymbol{m}^{(0)}}{\delta} + \frac{\boldsymbol{\mu}\boldsymbol{m}^{(1)}}{\delta} - \left[\frac{\boldsymbol{\mu}\boldsymbol{m}^{(0)}}{\delta}\right]^2 \tag{18}$$

where we have defined the vector $\boldsymbol{\mu} = (\mu_{f^{-1}(1)}, \ldots, \mu_{f^{-1}(4(n+1))})$ and $\mathbf{m}^{(j)} = \sum_m m^j p(m)$ the jth moment of the number of RNAs per cell. The vectors $\boldsymbol{m}^{(0)}$ is the steady-state probability distribution of the Markov chain $\boldsymbol{K}$ (i.e. $\boldsymbol{m}^{(0)}$ is a solution of equation (16)).

The vectors $\boldsymbol{m}^{(1)}$ is the solution of the equation

$$(\mathbf{K} - \mathbf{\Delta})\mathbf{m}^{(1)} + \boldsymbol{T}\mathbf{m}^{(0)} = 0. \tag{19}$$

(see equation 4 in Sánchez and Kondev [2008]).

4.3 Mean and variance of the number of RNAs as a function of the contact probability

In the enhancer-promoter model, the *contact probability* is the steady-state probability that the enhancer is in the close state. It can be directly calculated from the close rate and the far rate,

$$p_c = \frac{k_{close}}{k_{close} + k_{far}}. \tag{20}$$

From equation (20), we can express k_{close} in terms of k_{far} and p_c,

$$k_{close} = \frac{k_{far} p_c}{1 - p_c}. \tag{21}$$

We substitute equation (21) for k_{close} in equation (15) and obtain the mean and the variance of the number of RNA molecules as a function of the rate parameters k_{far}, k_{back}, $k_{forward}$, k_{on}^{basal}, k_{on}^{enh}, k_{off}, μ, the number of intermediate regulatory steps n, and the contact probability p_c.

5 Qualitative study of the transcriptional response to changes in contact probabilities

The fit of the k_{on} two-state model (see Section 3) shows that the mean and the cell-to-cell variability in number of mRNAs per cell can be explained by a two-state model in which the *on* rate depends on the contact probability between the enhancer and the promoter in a sigmoidal manner. However, the model of enhancer-promoter communication described in Section 4 can not in general be approximated by a two-state model. In this Section, we investigate for which parameters our mechanistic enhancer-promoter model reduces to an apparent two-state model in which the *on* rate, k_{on}^{app}, depends "sigmoidally" on contact probability.

5.1 Reduction of the enhancer-promoter model to an apparent two-state model

We apply the theory of aggregation of states in Markov chain with weak interaction Gaitsgori and Pervozvanskii [1975]. When enhancer-promoter interactions and intermediate regulatory steps kinetics are both faster than the promoter's intrinsic transcriptional dynamics, the Markov chain described by the chemical equation (15) can be separated in a fast Markov chain describing the transitions between the enhancer states and the promoter regime states, and an apparent (or slow) Markov chain describing the transitions between the promoter states (i.e. *on* and *off* states) and the number of mRNAs. The rates of the apparent chain are the weighted average of the rates across all the combinations of enhancer states (close, far) and promoter regime states $(1, \ldots, n+1)$. The weights are given by the steady state distribution of the fast chain evaluated at each combinations of states. Since the *on* rate only depends on promoter regime states (i.e. k_{on}^{basal} when the promoter is in the states $1, \ldots, n$, and k_{on}^{enh} when the promoter is in the state $n+1$), the *on* rate of the apparent chain is

$$
\begin{aligned}
k_{on}^{app} &= p_{enh} k_{on}^{enh} + (1 - p_{enh}) k_{on}^{basal} && (22)\\
&= k_{on}^{basal} + p_{enh}(k_{on}^{enh} - k_{on}^{basal}). && (23)
\end{aligned}
$$

where p_{enh} is the probability, at steady-state of the fast Markov chain, that the promoter is in the enhanced regime (i.e. in the state $n+1$). Since the *off* and the initiation rates do not depend on the enhancer state and neither on the promoter regime state, they are unchanged for the apparent chain (i.e. $k_{off}^{app} = k_{off}$ and $\mu^{app} = \mu$.) Theorem 1 in Gaitsgori and Pervozvanskii [1975] shows that when the difference of time scales is large enough (i.e. $k_{close}, k_{far}, k_{back}, k_{forward}$ are sufficiently larger than $k_{on}^{basal}, k_{on}^{enh}, k_{off}, \mu$), the marginal distribution of the promoter states in the steady-state of the full model is well approximated by the steady-state distribution of the apparent chain.

In conclusion, when enhancer-promoter interactions and intermediate regulatory steps kinetics are both faster than the promoter's intrinsic transcriptional dynamics, the full model of enhancer-promoter communication reduces to an apparent two-state model in which the *on* rate is given by equation (22). We now ask for which parameters this apparent *on* rate depends sigmoidally on contact probability.

5.2 Sigmoidallity of the apparent *on* rate

We first focus on the low sensitivity of the apparent *on* rate at high contact probability. We search parameters values for which the apparent *on* rate "plateaus" at high contact probability.

In order to select those parameters, we first note that in the apparent two-state model, contact probability affects k_{on}^{app} by modulating the probability that the promoter is in the enhanced regime (see equation (23)). Thus the desire parameters are the ones for which the enhanced regime probability p_{enh} is poorly sensitive to change in contact probability at high contact probabilities. The enhanced regime probability, p_{enh}, is calculated from the steady state probability distribution $\mathbf{v}$ of the Markov chain $\boldsymbol{K}$ (see equation (16)) by summing its elements that correspond to the enhanced regime (i.e. $(i, j, n+1)$ for $i = 1, 2$ and $j = 1, 2$) which corresponds to

$$p_{enh} = \mathbf{e}^{enh}\mathbf{v}, \tag{24}$$

where $\mathbf{e}^{enh}$ is the vector of length $4(n+1)$ whose elements $e_{f(i,j,k)}^{enh} = 1$ are 1 if $k = n+1$ and 0 elsewhere.

The sensitivity of $p_{enh}(p_c)$ at high contact probability can be assessed by calculating its first and second derivatives at contact probability 1. The sensitivity is the lowest when both the first and second derivatives are the lowest. At contact probability 1, the first and second derivatives of the enhanced regime probability are given by

$$\frac{\partial p_{enh}}{\partial p_c}(1) = \frac{u^n}{(n + (n-1)u + \cdots + u^{n-1})^2} \tag{25}$$

and

$$\frac{\partial^2 p_{enh}}{\partial p_c^2}(1) = \frac{z p_1(u) + p_2(u)}{z(1 + u + \cdots + u^n)^3} \tag{26}$$

where $z = \frac{k_{far}}{k_{back}}$ and $u = \frac{k_{forward}}{k_{back}}$, p_1 is a polynomial in variable u of degree $3n-1$, and p_2 is a polynomial in variable u of degree $3n$. Equations (25) and (26) were calculated for different value of n using the *symbolic toolbox* in *Matlab* (version 2019b). We deduce from equations (25) and (26) that sensitivity is minimised when u and z are large, which means when the ratio $\frac{k_{forward}}{k_{back}}$ and the ratio $\frac{k_{far}}{k_{back}}$ are large. Thus, the rate parameters should be such that memory is long, i.e. the promoter remains in the enhanced regime much longer than the average duration of an interaction ($\frac{k_{far}}{k_{back}} >> 1$), and the intermediate regulatory steps are fast ($\frac{k_{forward}}{k_{back}} >> 1$).

The condition $\frac{k_{far}}{k_{back}} >> 1$ implies that the timescales of the enhancer-promoter interactions and the regulatory steps kinetics are decoupled. Hence, we can apply the theory of aggregation of states in Markov chain with weak interaction Gaitsgori and Pervozvanskii [1975] to the Markov chain describing the transition between enhancer states and promoter regime states. We denote this chain by $\mathbf{K}_f$. In this way, we can deduce an approximation of the enhanced regime probability p_{enh} which is valid in the limit $\frac{k_{far}}{k_{back}} >> 1$. We separate the $\mathbf{K}_f$ chain in a "super" fast Markov chain, $\mathbf{K}_{ff}$, describing the transitions between the enhancer states (i.e. Close and Far), and a slower Markov chain, $\mathbf{K}_{fs}$, describing the transitions between the promoter regime states (i.e. $1, \ldots, n+1$). The forward and backward rates of the slower chain $\mathbf{K}_{fs}$ are the weighted average of the forward and backward rates across all the enhancer states. The weights are the corresponding values of the steady state distribution of the super fast chain $\mathbf{K}_{ff}$. Since only the forward rate depends on the enhancer state (i.e. $k_{forward}$ when the enhancer is close, and 0 when the enhancer is far), the forward rate of the slower chain is

$$k_{forward}^{fs} = p_c k_{forward}. \tag{27}$$

and the backward rate of the slower chain is k_{back}, yielding the transition matrix

$$\mathbf{K}_{fs} = \begin{pmatrix} -\gamma_+ & \gamma_+ & & & \\ \gamma_- & -(\gamma_+ + \gamma_-) & \gamma_+ & & \\ & & & & \\ & \ddots & \ddots & \ddots & \\ & & & & \\ & & \gamma_- & -(\gamma_+ + \gamma_-) & \gamma_+ \\ & & & \gamma_- & -\gamma_- \end{pmatrix} \tag{28}$$

where $\gamma_+ = p_c k_{forward}$ and $\gamma_- = k_{back}$. By solving the equation $\boldsymbol{w}^T \mathbf{K}_{fs} = 0$, we obtain the steady-state distribution of the slower chain $\mathbf{K}fs$,

$$w_k = \frac{(1 - \gamma_+/\gamma_-)(\gamma_+/\gamma_-)^{k-1}}{1 - (\gamma_+/\gamma_-)^{n+1}}, \text{ for } k = 1, \ldots, n+1 \tag{29}$$

Theorem 1 in Gaitsgori and Pervozvanskii [1975] shows that when the difference of time scales is large enough (i.e. k_{close}, k_{far} are sufficiently larger than $k_{back}, k_{forward}$), the marginal distribution of the promoter regime states in the steady-state of the chain $\boldsymbol{K}_f$ is well approximated by the steady-state distribution of the slower chain $\mathbf{K}_{fs}$. In this limit case, the enhancer probability p_{enh} is thus given by w_{n+1}, i.e.

$$p_{enh} = \frac{(1 - p_c\beta)(p_c\beta)^n}{1 - (p_c\beta)^{n+1}} \tag{30}$$

where $\beta = k_{forward}/k_{back}$. This function is sigmoidal-like only if $n > 1$ and if $\beta < 1$.

In conclusion, the two-state behaviour observed in the smFISH data and sigmoidal-lilke shape of the mean transcriptional response to change in contact probability can be recapitulated with our enhancer-promoter model when the timescales of enhancer-promoter interactions are faster than those of the intermediate regulatory steps, and both are faster than the promoter's intrinsic bursting dynamics (i.e. $k_{close}, k_{far} >> k_{back}, k_{forward} >> k_{on}^{basal}, k_{on}^{enh}, k_{off}, \mu$), there are more than 1 regulatory step (i.e. $n > 1$), and forward reaction are favoured over backward reactions. In this scenario, the marginal steady-state-distribution of the promoter states and mRNA number of the full enhancer-promoter model is well approximated by the steady-state distribution of an apparent two-state model with *off* rate k_{off}, initiation rate μ, and *on* rate

$$k_{on}^{app} = k_{on}^{basal} + \frac{(1 - p_c\beta)(p_c\beta)^n}{1 - (p_c\beta)^{n+1}} \left(k_{on}^{enh} - k_{on}^{basal}\right). \tag{31}$$

6 Enhancer-promoter model fitting

Analysis of the enhancer-promoter model (see Section 5) concludes that the model could reproduce qualitatively the observed data when the timescales of enhancer-promoter interactions are faster than those of the intermediate regulatory steps, and both are faster than the promoter's intrinsic bursting dynamics, there are more than 1 regulatory step (i.e. $n > 1$), and forward reaction are favoured over backward reactions. In this scenario we shown that the model is well approximated by an apparent variable two state model with the *on* rate described in equation (31). We thus fit this apparent two-state model to the data. In the sequel, all the rates are expressed in unit of 1 over the mean life time of a mRNA molecule (i.e. we set $\delta = 1$).

6.1 Full SCR data set

We fit the apparent two-state model simultaneously to the mean eGFP levels measured in individual cell lines and to the distributions of RNA numbers measured by smRNA FISH in 6 cell lines where the full-length Sox2 control region (SCR) was located at different distances from the promoter. The fitted parameters are the number of regulatory steps, n, the ratio between the forward and backward rates of the regulatory steps, β, the basal and enhanced *on* rates, k_{on}^{basal} and k_{on}^{enh}, and the *off* and initiation rates, k_{off} and μ. We follow the same procedure described in Section 3 for the variable k_{on} two-state model. Since the number of regulatory steps n is an integer parameter, we maximise the total log likelihood function LL_{tot} for each value of n separately. For the parameter n, we set a lower bound of 1 and an upper bound of 10. For all the other parameters (i.e. $\beta, k_{on}^{basal}, k_{on}^{enh}, k_{off}, \mu$), we set a lower bound of 0 and an upper bound of 1000 for the parameters $k_{on}^{basal}, k_{on}^{enh}, k_{off}, \mu$ and an upper bound of 100 for the parameter β. We ensured that the best fit parameters found were not at the boundaries. For all the maximisations we use a global search approach. Specifically, we use the Matlab function *MultiStart* in the *Global Optimization* toolbox. All codes were written in Matlab (version 2019b) and are available at `https://github.com/gregroth/Zuin_Roth_2021`.

6.2 Truncated SCR data set

In the apparent two-state model, the enhancer can affect transcription through 2 parameters, namely β which determines the ratio between the forward and the backward rates of the regulatory steps and/or k_{on}^{enh} the *on* rate in the enhanced regime. To determine which of those parameters does the enhancer strength affect, we compare 3 versions of the apparent two-state model in which the parameter β (model 1) or k_{on}^{enh} (model 2), or both (model 3) are free parameters and the other ones are fixed to the best fit values $\boldsymbol{\theta}^*$ obtained for the full-length SCR data set (see Section 6.1). We fit each model to the binned mean number of RNA molecule inferred from the eGFP+ cell lines with the truncated version of the SCR. For each model we calculate the maximum log likelihood i.e.

$$\ell_1 = \max_{\{\theta_1\}} LL_{mean}(\theta_1, \theta_2^*, \ldots, \theta_6^*) \quad (32)$$

$$\ell_2 = \max_{\{\theta_3\}} LL_{mean}(\theta_1^*, \theta_2^*, \theta_3, \theta_4^*, \theta_5^*, \theta_6^*) \quad (33)$$

$$\ell_3 = \max_{\{\theta_1,\theta_3\}} LL_{mean}(\theta_1, \theta_2^*, \theta_3, \theta_4^*, \theta_5^*, \theta_6^*) \quad (34)$$

For each model, we calculate the maximum log-likelihood ratio

$$\lambda_j = -2(\ell_j - \ell^*). \quad (35)$$

where $\ell^* = \max_{\{\boldsymbol{\theta}\}} LL_{mean}(\boldsymbol{\theta})$ is the maximum log likelihood over all the parameters. Under the null-hypothesis that the data can be explained by the jth model, the ratio λ_j converges to a chi squared distribution with 4 degrees of freedom Wilks [1938]. Only the model with k_{on}^{enh} as free parameter was able to account for the data (p-value $= 0.4967$). The models with β and k_{on}^{basal} as free parameters were not able to reproduce the data (p-values $< .00001$).

References

V. G. Gaitsgori and A. A. Pervozvanskii. Aggregation of states in a Markov chain with weak interaction. *Cybernetics*, 11(3):441–450, 1975. ISSN 0011-4235.

Ankit Gupta, Jan Mikelson, and Mustafa Khammash. A finite state projection algorithm for the stationary solution of the chemical master equation. *The Journal of Chemical Physics*, 147(15):154101, 2017.

Brian Munsky, Gregor Neuert, and Alexander van Oudenaarden. Using Gene Expression Noise to Understand Gene Regulation. *Science*, 336(6078):183–187, 04 2012. ISSN 0036-8075.

Y. Pawitan. *In All Likelihood: Statistical Modelling and Inference Using Likelihood.* Oxford Science Publications, Clarendon Press, Oxford., New York, New York, 2001.

J Peccoud and B Ycart. Markovian modelling of gene product synthesis. *Theoretical Population Biology*, pages 1 – 13, 08 1995.

Álvaro Sánchez and Jané Kondev. Transcriptional control of noise in gene expression. *Proceedings of the National Academy of Sciences*, 105(13):5081–5086, 2008.

S. S. Wilks. The Large-Sample Distribution of the Likelihood Ratio for Testing Composite Hypotheses. *The Annals of Mathematical Statistics*, 9(1):60 – 62, 1938.

Rate	Expression	From	To
Hyper state transition rates			
$k_{(1,j,k;2,j,k)}$ for $j = 1, 2$, $k = 1, \ldots, n+1$	k_{far}	close	far
$k_{(2,j,k;1,j,k)}$ for $j = 1, 2$, $k = 1, \ldots, n+1$	k_{close}	far	close
$k_{(i,1,k;i,2,k)}$ for $k = 1, \ldots, n$	k_{on}^{basal}	off/low	on/low
$k_{(i,1,n+1;i,2,n+1)}$ for $i = 1, 2$	k_{on}^{enh}	off/high	on/high
$k_{(i,2,k;i,1,k)}$ for $k = 1, \ldots, n+!$	k_{off}	on/low	off/low
$k_{(1,j,k;1,j,k+1)}$ for $j = 1, 2$, $k = 1, \ldots, n$	$k_{forward}$	close/r_k	close/r_{k+1}
$k_{(2,j,k;2,j,k+1)}$ for $j = 1, 2$, $k = 1, \ldots, n$	0	far/r_k	far/r_{k+1}
$k_{(i,j,k;i,j,k-1)}$ for $j = 1, 2$, $k = 2, \ldots, n+1$	k_{back}	r_k	r_{k-1}
Initiation rates			
$\mu_{i,2,k}$ for $i = 1, 2$, $k = 1, \ldots, n+1$	μ	on	on+1 RNA

Table 1: Transition rates used in equations (11) and (15). All the rates $\mu_{(i,j,k)}$ and $k_{(i,j,k;\bar{i},\bar{j},\bar{k})}$ that are not described in the table have value equal to 0.

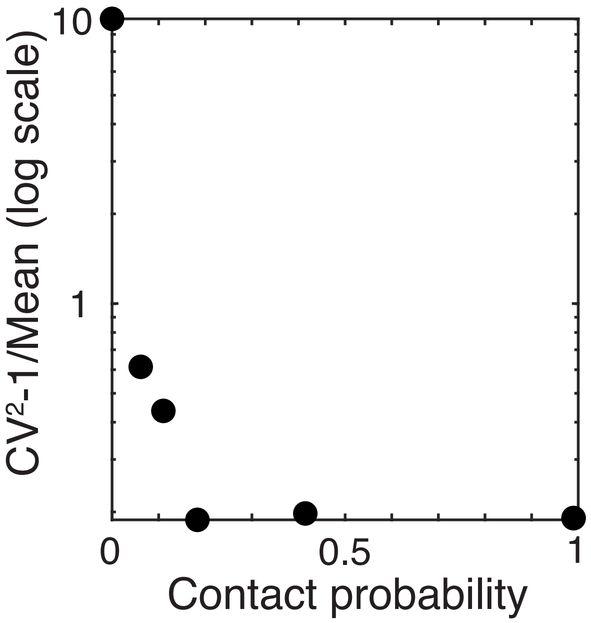

Figure 1: Difference between the squared coefficient of variation and the inverse of the mean, Δ, plotted against contact probabilities between the ectopic Sox2 promoter and the locations of SCR in cell the lines shown in Fig. 2C-D ($\Delta = 0$ for a Poisson distribution).

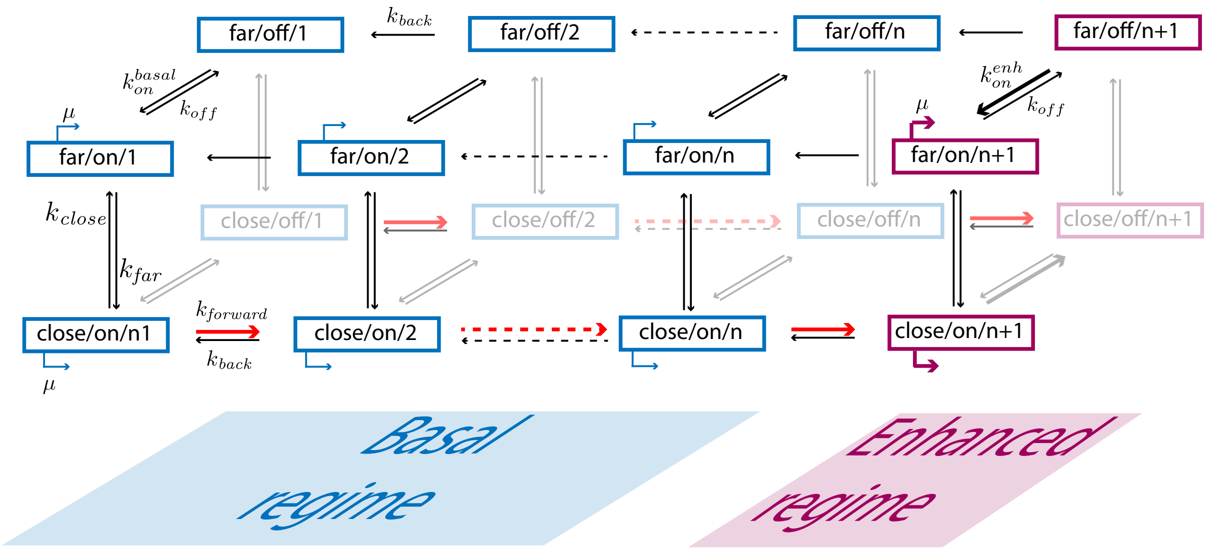

Figure 2: Scheme of the enhancer-promoter model. For simplicity every rate is indicated only once in similar reactions. Opacity differences are only intended to increase the clarity of the figure and do not relate to properties of the states themselves.

Gating Strategy for Nonlinear control of transcription through enhancer-promoter interactions

Content

1. Gate Strategy: Single Cell FACS sort of GFP+ cell lines from PiggyBac-enhancer Founder lines
2. Gate Strategy: Single Cell FACS sort of GFP+ cell lines from Promoter only Founder line
3. Gate Strategy: Single Cell FACS sort of GFP+ cell lines from PiggyBac-enhancer Founder line using the Standard Gate Strategy on eGFP levels (Extended Data Fig. 1l, top panel)
4. Gate Strategy: Single Cell FACS sort of GFP+ cell lines from PiggyBac-enhancer Founder line using a less stringent Gate Strategy on eGFP level (Extended Data Fig. 1l, bottom panel)
5. Gate Strategy: FACS sort of pools of cells for tagmentation-based mapping of PiggyBac-enhancer insertions (Extended Data Fig. 1m)

Gate Strategy: Single Cell FACS sort of GFP+ cell lines from PiggyBac-enhancer Founder lines

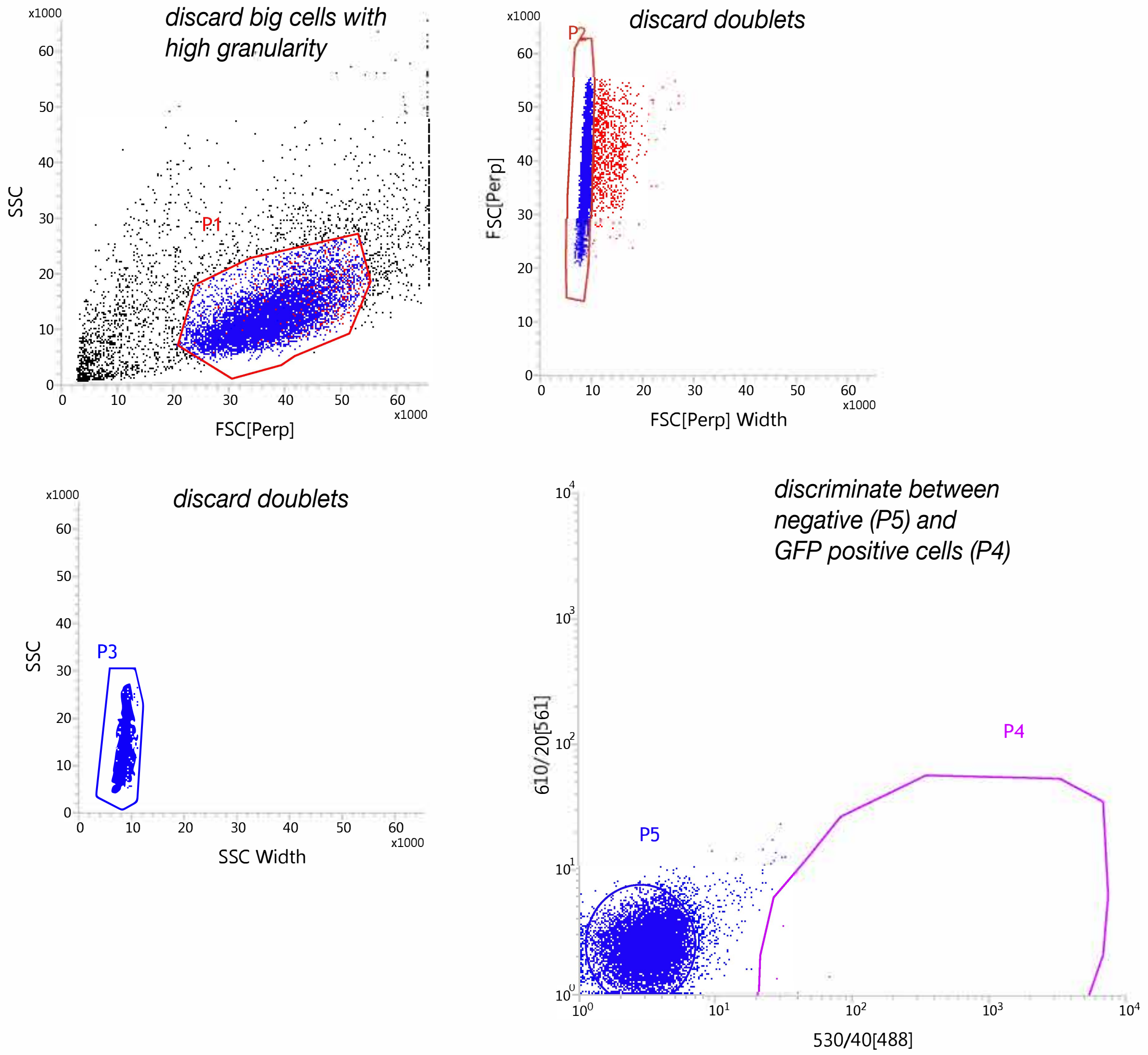

Statistics:

Populations	Events	% Total	% Parent	530/40[... Mean	610/20[... Mean
All Events	14,668	100.00%	####	5	3
P1	11,384	77.61%	77.61%	4	3
P2	10,000	68.18%	87.84%	4	3
P3	10,000	68.18%	100.00%	4	3
P4	4	0.03%	0.04%	41	2
P5	9,335	63.64%	93.35%	3	3

Gating Strategy:

FSC / SSC: to discard big cells with high granularity;
FSC-W / FSC: to discard doublets;
SSC-W / SSC: to discard doublets;
530/40[488] / 610/20[561]: to discriminate between negative (P5) and GFP positive cells (P4)

Gate Strategy: Single Cell FACS sort of GFP+ cell lines from Promoter only Founder line

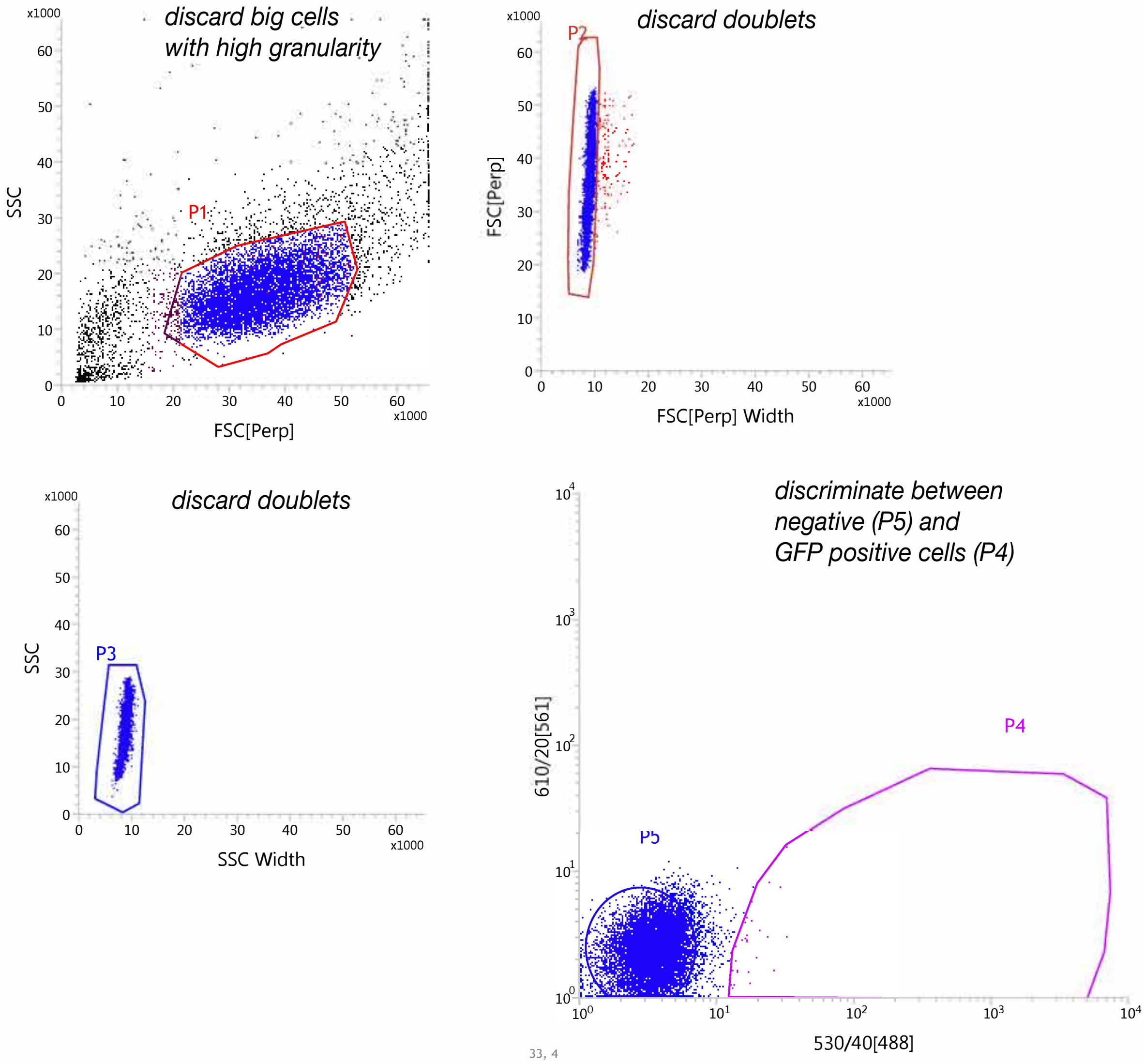

Statistics:				530/40[...	610/20[...
Populations	Events	% Total	% Parent	Mean	Mean
All Events	12,668	100.00%	####	4	3
P1	10,186	80.41%	80.41%	4	3
P2	10,000	78.94%	98.17%	4	3
P3	10,000	78.94%	100.00%	4	3
P4	30	0.24%	0.30%	18	3
P5	9,160	72.31%	91.60%	4	3

Gating Strategy:

FSC / SSC: to discard big cells with high granularity;
FSC-W / FSC: to discard doublets;
SSC-W / SSC: to discard doublets;
530/40[488] / 610/20[561]: to discriminate between negative (P5) and GFP positive cells (P4)

Gate Strategy: Single Cell FACS sort of GFP+ cell lines from PiggyBac-enhancer Founder line using the Standard Gate Strategy on eGFP levels (Extended Data Fig. 1l, top panel)

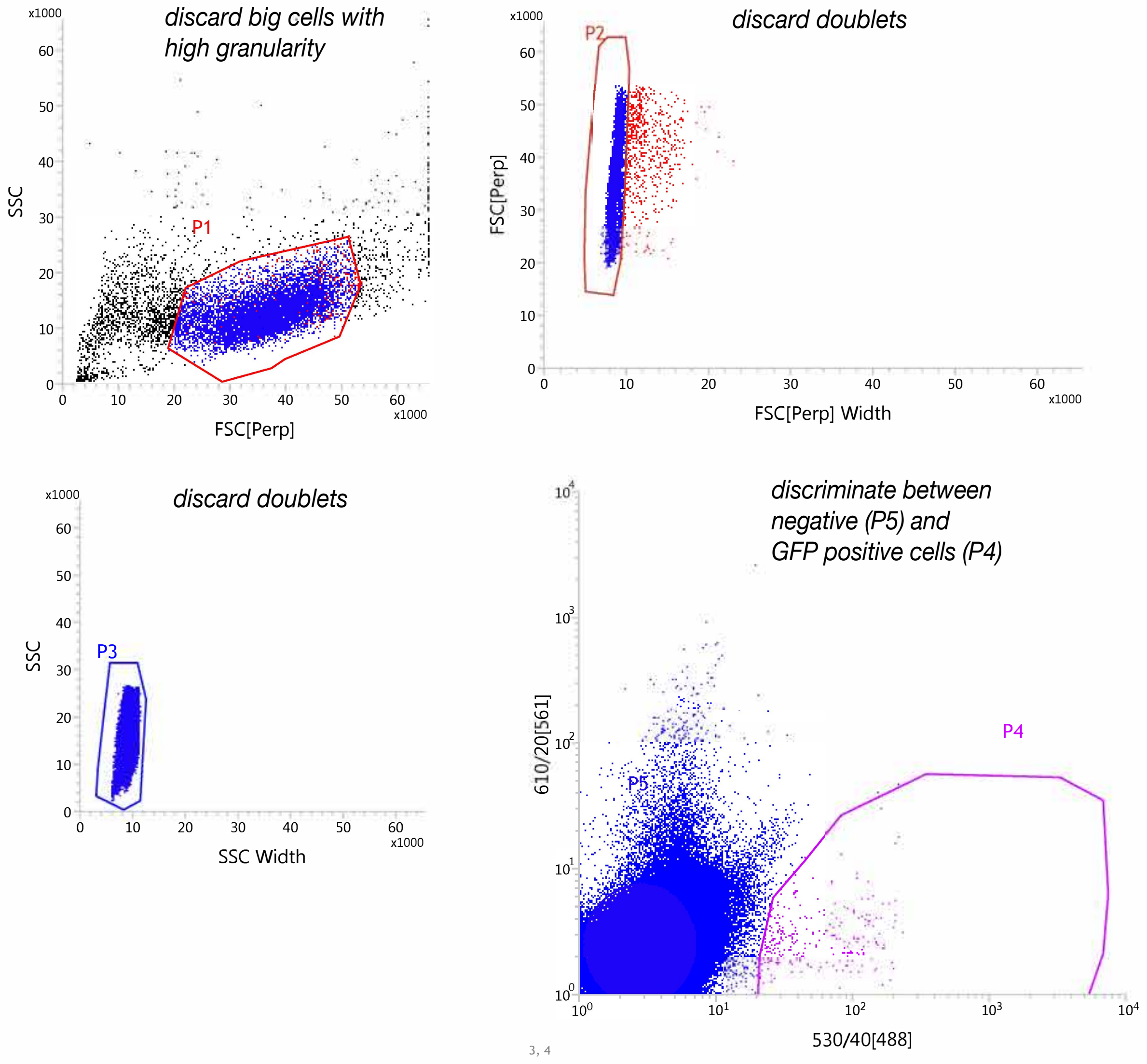

Statistics:

Populations	Events	% Total	% Parent	530/40[... Mean	610/20[... Mean
All Events	695,588	100.00%	####	5	4
P1	546,944	78.63%	78.63%	5	3
P2	500,000	71.88%	91.42%	4	3
P3	500,000	71.88%	100.00%	4	3
P4	295	0.04%	0.06%	63	3
P5	432,608	62.19%	86.52%	4	3

Gating Strategy:

FSC / SSC: to discard big cells with high granularity;
FSC-W / FSC: to discard doublets;
SSC-W / SSC: to discard doublets;
530/40[488] / 610/20[561]: to discriminate between negative (P5) and GFP positive cells (P4)

Gate Strategy: Single Cell FACS sort of GFP+ cell lines from PiggyBac-enhancer Founder line using a less stringent Gate Strategy on eGFP level (Extended Data Fig. 1l, bottom panel)

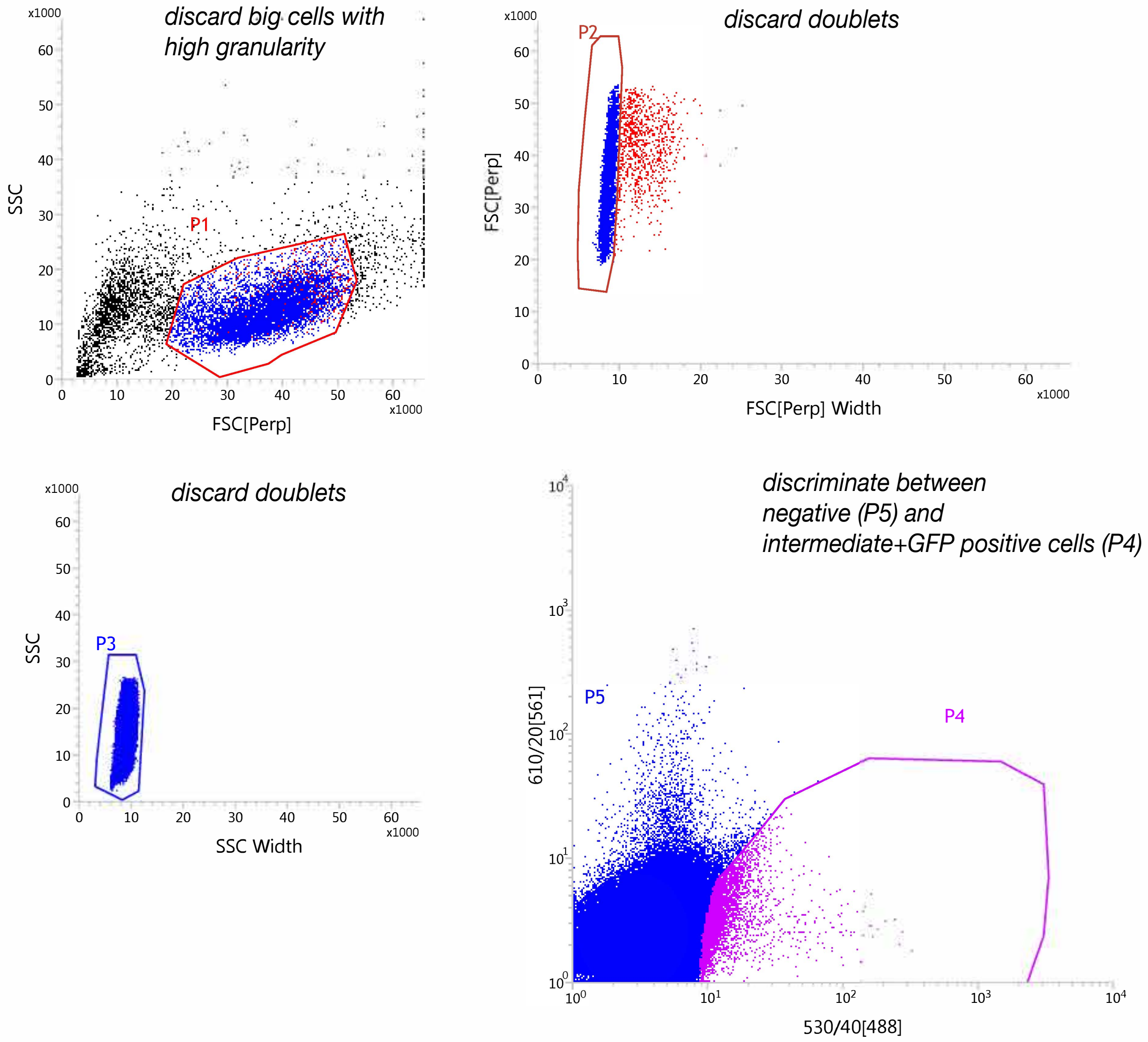

Statistics:

Populations	Events	% Total	% Parent	530/40[... Mean	610/20[... Mean
All Events	791,303	100.00%	####	5	4
P1	578,632	73.12%	73.12%	5	3
P2	500,000	63.19%	86.41%	5	3
P3	500,000	63.19%	100.00%	5	3
P4	4,779	0.60%	0.96%	14	4
P5	429,497	54.28%	85.90%	4	3

Gating Strategy:

FSC / SSC: to discard big cells with high granularity;
FSC-W / FSC: to discard doublets;
SSC-W / SSC: to discard doublets;
530/40[488] / 610/20[561]: to discriminate between negative (P5) and intermediate+GFP positive cells (P4)

Gate Strategy: FACS sort of pools of cells for tagmentation-based mapping of PiggyBac-enhancer insertions (Extended Data Fig. 1m)

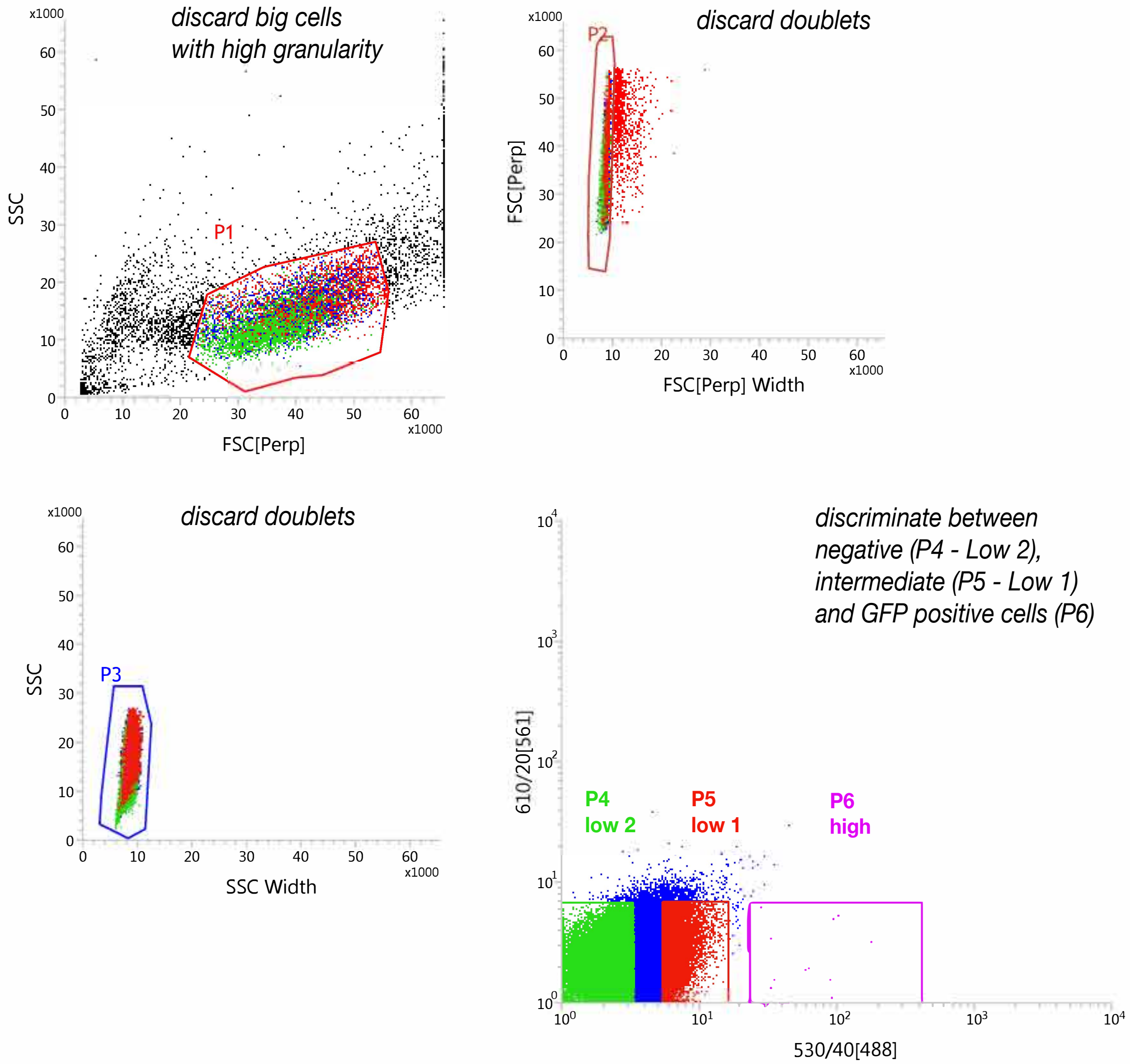

Statistics:

Populations	Events	% Total	% Parent	530/40[... Mean	610/20[... Mean
All Events	156,102	100.00%	####	5	3
P1	114,327	73.24%	73.24%	4	3
P2	96,873	62.06%	84.73%	4	3
P3	96,873	62.06%	100.00%	4	3
P4	39,812	25.50%	41.10%	2	2
P5	15,132	9.69%	15.62%	6	3
P6	13	0.01%	0.01%	65	3

Gating Strategy:

FSC / SSC: to discard big cells with high granularity;
FSC-W / FSC: to discard doublets;
SSC-W / SSC: to discard doublets;
530/40[488] / 610/20[561]: to discriminate between negative (P4 - low 2), intermediate (P5 - low 1) and GFP positive cells (P6 - high)
