# Supplementary material for: Nonlinear control of transcription through enhancer–promoter interactions
Source: Nature. 2022 Apr 13;604(7906):571–7. doi: 10.1038/s41586-022-04570-y (PMC9021019; doi:10.1038/s41586-022-04570-y)
Supplement: Supplementary file 2 — Reporting Summary [file 41586_2022_4570_MOESM2_ESM.pdf]

nature research

Corresponding author(s): Luca Giorgetti
Last updated by author(s): Jan 12, 2022

Reporting Summary

Nature Research wishes to improve the reproducibility of the work that we publish. This form provides structure for consistency and transparency in reporting. For further information on Nature Research policies, see our Editorial Policies and the Editorial Policy Checklist.

Statistics

For all statistical analyses, confirm that the following items are present in the figure legend, table legend, main text, or Methods section.

n/a	Confirmed	
☐	☒	The exact sample size (n) for each experimental group/condition, given as a discrete number and unit of measurement
☐	☒	A statement on whether measurements were taken from distinct samples or whether the same sample was measured repeatedly
☐	☒	The statistical test(s) used AND whether they are one- or two-sided<br>*Only common tests should be described solely by name; describe more complex techniques in the Methods section.*
☐	☒	A description of all covariates tested
☐	☒	A description of any assumptions or corrections, such as tests of normality and adjustment for multiple comparisons
☐	☒	A full description of the statistical parameters including central tendency (e.g. means) or other basic estimates (e.g. regression coefficient) AND variation (e.g. standard deviation) or associated estimates of uncertainty (e.g. confidence intervals)
☐	☒	For null hypothesis testing, the test statistic (e.g. F, t, r) with confidence intervals, effect sizes, degrees of freedom and P value noted<br>*Give P values as exact values whenever suitable.*
☒	☐	For Bayesian analysis, information on the choice of priors and Markov chain Monte Carlo settings
☒	☐	For hierarchical and complex designs, identification of the appropriate level for tests and full reporting of outcomes
☒	☐	Estimates of effect sizes (e.g. Cohen's d, Pearson's r), indicating how they were calculated

Our web collection on statistics for biologists contains articles on many of the points above.

Software and code

Policy information about availability of computer code

Data collection	Zeiss Axion Observer Z1 was used for microscopy image collection (RNA FISH), BD LSRII SORP Analyser + HTS was used for acquiring GFP intensity, BD Influx cell sorter was used for the FACS
Data analysis	Matlab (version 2019b), global optimisation toolbox (Matlab), symbolic toolbox (Matlab), minimap2 (v. 2.17-r941), Snakemake (v. 3.13.3), IGV (v. 2.9.4), HiC-Pro (v. 2.11.4), ChromHMM (v.1.14), bwa (v. 0.7.17), Biostrings (v. 2.58.0), FlowJo (v. 10.6.2), Knime (3.7.2), Fiji (v. 2.0), TrackMate (v. 6.0.0), Pandas (v. 1.1.0), python 2.7, QuasR 1.34.0, STAR 2.5.0a. Flow Cytometry: BD FACSDiva™ Software. FACS: BD FACS™ Software 1.2.0.142. Custom codes can be found in https://github.com/zhanyinx/Zuin_Roth_2021, https://github.com/gregroth/Zuin_Roth_2021 and https://github.com/vansteensellab/tagmap_hopping/tree/giorgetti

For manuscripts utilizing custom algorithms or software that are central to the research but not yet described in published literature, software must be made available to editors and reviewers. We strongly encourage code deposition in a community repository (e.g. GitHub). See the Nature Research guidelines for submitting code & software for further information.

Data

Policy information about availability of data

All manuscripts must include a data availability statement. This statement should provide the following information, where applicable:

- Accession codes, unique identifiers, or web links for publicly available datasets
- A list of figures that have associated raw data
- A description of any restrictions on data availability

All cHi-C, Oxford Nanopore, tagmentation and population-based splinkerette PCR sequencing fastq files generated in this study have been uploaded to the Gene Expression Omnibus (GEO) under accession GSE172257 (https://www.ncbi.nlm.nih.gov/geo/query/acc.cgi?acc=GSE172257). The following public databases were

used: BSgenome.Mmusculus.UCSC.mm9, TxDb.Mmusculus.UCSC.mm9.knownGene.

Field-specific reporting

Please select the one below that is the best fit for your research. If you are not sure, read the appropriate sections before making your selection.

- [x] Life sciences
- [ ] Behavioural & social sciences
- [ ] Ecological, evolutionary & environmental sciences

For a reference copy of the document with all sections, see nature.com/documents/nr-reporting-summary-flat.pdf

Life sciences study design

All studies must disclose on these points even when the disclosure is negative.

Sample size	For cHi-C data we used 2 biological replicates. For RNA FISH we used 3 replicates for each cell line. The flow cytometry measurements were performed in 3 biological replicates. We did not apply statistical methods to pre-determine sample size and followed the general standard practice in the field. Number of replicate experiments is indicated in the legends.
Data exclusions	In enhancer mobilisation experiments, enhancer insertions within the transgene itself were omitted as they disrupt the sequence of the transgene and eGFP levels cannot be compared with other enhancer genomic positions.
Replication	RNA FISH was performed in triplicates. Flow Cytometry measurements were performed in triplicates. cHi-C was performed in duplicates. Mobilization experiments in ΔΔCTCF background were performed twice. The other mobilisation experiments were performed once. Each mobilisation experiments lead to hundreds different cell lines which can be interpreted as replicates.
Randomization	Randomization is not applicable to this study as we used only cell lines and no human or animal subjects were used in this study
Blinding	RNA FISH experiments and analysis were performed in a blinded manner. Blinding was not necessary for the other experiments since the results are quantitative and did not require subjective judgment or interpretation.

Reporting for specific materials, systems and methods

We require information from authors about some types of materials, experimental systems and methods used in many studies. Here, indicate whether each material, system or method listed is relevant to your study. If you are not sure if a list item applies to your research, read the appropriate section before selecting a response.

Materials & experimental systems

n/a	Involved in the study	
x		Antibodies
	x	Eukaryotic cell lines
x		Palaeontology and archaeology
x		Animals and other organisms
x		Human research participants
x		Clinical data
x		Dual use research of concern

Methods

n/a	Involved in the study	
x		ChIP-seq
	x	Flow cytometry
x		MRI-based neuroimaging

Eukaryotic cell lines

Policy information about cell lines

Cell line source(s)	All cell lines are based on E14 mouse embryonic stem cells (mESCs) provided by Edith Heard laboratory, EMBL, Heidelberg
Authentication	Cell lines have been recurrently used by the authors in previous studies and therefore have not been authenticated.
Mycoplasma contamination	Cells were tested for mycoplasma contamination once a month and no contamination was detected.
Commonly misidentified lines (See ICLAC register)	No commonly misidentified lines were used.

Flow Cytometry

Plots

Confirm that:

- [x] The axis labels state the marker and fluorochrome used (e.g. CD4-FITC).
- [x] The axis scales are clearly visible. Include numbers along axes only for bottom left plot of group (a 'group' is an analysis of identical markers).
- [x] All plots are contour plots with outliers or pseudocolor plots.
- [x] A numerical value for number of cells or percentage (with statistics) is provided.

Methodology

Sample preparation	Flow Cytometry: Cells were harvested with Accutase and re-suspend in E14 medium (supplemented with 2i in the case of the remobilization experiment). FACS: cells were harvested with Accutase and re-suspend in E14 medium with only 3% of FCS, 100 µg/m primocin (InvivoGen, ant-pm-1) and 10uM ROCK inhibitor (STEMCELL Technologies, Y-27632).
Instrument	Flow Cytometry: BD LSRII SORP Analyser (Becton Dickinson) for transfection efficiency and enhancer reporter assay and BD LSRII SORP Analyser + HTS for enhancer mobilization assay. FACS: BD Influx cell sorter (Becton Dickinson)
Software	Flow Cytometry: BD FACSDiva™ Software. FACS: BD FACS™ Software 1.2.0.142
Cell population abundance	Flow Cytometry: 10000 cells were acquired for enhancer mobilization assay and >10000 cells were acquired for transfection efficiency and enhancer reporter assay. FACS: single cells sort was performed by sorting typically six 96-well plates for each founder line.
Gating strategy	Flow Cytometry gating: FSC/SSC to discard big cells with high granularity; SCC-W/SCC-H to discard doublets; FSC-A/Dapi to discard dead cells; GFP/histogram to quantify GFP intensity. FACS: FSC/SSC to discard big cells with high granularity; FSC-W/FSC: to discard doublets; SSC-W/SSC: to discard doublets; 530/40[488]/610/20[561]: to discriminate between negative and GFP positive cells.

- [x] Tick this box to confirm that a figure exemplifying the gating strategy is provided in the Supplementary Information.
